# Glucose metabolism is linked to the inflammatory status of macrophages

**DOI:** 10.1186/1753-6561-6-S3-P62

**Published:** 2012-06-01

**Authors:** Amy R Johnson, Alex J Freemerman, E Dale Abel, Jeffery Rathmell, Liza Makowski

**Affiliations:** 1 Department of Nutrition, Gillings School of Global Public Health, University of North Carolina at Chapel Hill, Chapel Hill, NC, 25799, USA; 2 Division of Endocrinology, Metabolism and Diabetes and Program in Molecular Medicine, University of Utah, Salt Lake City, UT 84132 USA; 3 Department of Pharmacology and Cancer Biology, Department of Immunology, Sarah W. Stedman Center for Nutrition and Metabolism, Duke University, Durham, NC 27708 USA

## Background

Macrophages infiltrate adipose tissue at the onset of weight gain and directly contribute to adipose inflammation, insulin resistance, and obesity [1]. The type of fuel substrate utilized by macrophages is central to the formation of obesity, a global epidemic [2]. Our goal is to understand the role of macrophage glucose metabolism in the promotion of inflammation and insulin resistance during high fat diet-induced obesity. We hypothesize that macrophages with *blunted or elevated* glucose metabolism will display *limited or exaggerated* immune responses, and modulate susceptibility to insulin resistance and obesity, respectively.

## Materials and methods

GLUT1 is the glucose transporter expressed by macrophages [3]. We manipulated macrophage glucose metabolism using GLUT1 over-expression and deletion techniques *in vitro*, *ex vivo*, and *in vivo. In vitro* studies involved over-expression of GLUT1 in RAW264.7 cells. A high fat diet-induced obesity model involving a novel macrophage-specific *Glut1* knockout mouse (*Glut1MΦ^-/-^*) was used to assess total body weight, glucose tolerance, tissue histological alterations, and gene expression changes resulting from *Glut1* deletion. Bone marrow-derived macrophages (BMDMs), isolated from *Glut1MΦ^-/-^mice* fed a control diet, were used for measures of polarization, and glucose uptake and metabolism.

## Results

GLUT1 over-expression resulted in elevated glucose uptake and metabolism, as well as a hyper-inflammatory state characterized by elevated secretion of MCP-1 and PAI-1, all of which could be blunted with a pharmacologic inhibitor of glycolysis. Preliminary data suggests that *Glut1MΦ^-/-^* mice fed a high fat diet were resistant to obesity, remained normoglycemic and demonstrated blunted inflammation in liver and adipose. *Glut1MΦ^-/-^* BMDMs were viable, but metabolized less glucose at baseline and after LPS stimulation.

## Conclusions

The capacity to use glucose as a fuel is correlated to the inflammatory status of macrophages which likely plays an integral role in the promotion of obesity-related insulin resistance. Possible mechanisms linking glucose metabolism to inflammation are being investigated. Understanding macrophage glucose metabolism and inflammation will identify metabolic and/or signaling pathways that will serve as novel therapeutic targets in the treatment of diabetes and obesity.
